# Real-world patient experience with medicinal cannabis use for symptom management in an Australian advanced cancer setting: a mixed method, cohort study using the theory of planned behaviour framework

**DOI:** 10.1007/s00520-024-09013-0

**Published:** 2024-11-15

**Authors:** Jennifer A. Ong, Un Cheng Lo, Hala Musa, Jeffery Li, Janet Gaon, Judith Lacey, Janet M. Y. Cheung, Michael Soriano

**Affiliations:** 1 School of Pharmacy, Faculty of Medicine and Health, University of Sydney, Camperdown, NSW Australia; 2 Department of Pharmacy, Chris O’Brien Lifehouse, Camperdown, NSW Australia; 3 Department of Supportive Care, Chris O’Brien Lifehouse, Camperdown, NSW Australia; 4 Clinical School of Medicine, University of Sydney, Camperdown, NSW Australia; 5 NICM Health Research Institute, Western Sydney University, Westmead, NSW Australia

**Keywords:** Medicinal cannabis, Medication hesitancy, Medication adherence, Theory of planned behaviour

## Abstract

**Purpose:**

Patient hesitancy to use MC due to the fear of negative social implications leads to intentional non-adherence and compromises therapeutic outcomes. Hence, we aimed to determine the rate of patient adherence to MC and explore factors influencing patient MC use.

**Methods:**

Demographic and quantitative data related to MC usage were extracted from medical records for patients prescribed MC at a single cancer centre in metropolitan Sydney. Qualitative data was generated from semi-structured interviews. Interview guides were developed based on the Theory of Planned Behaviour (TBP) domains (i.e. *Attitudes*, *Subjective Norms*, *Behavioural Intention* and *Perceived Behavioural Control*) to elucidate themes influencing MC use. A mixed method approach involving triangulation of quantitative and qualitative methods was used for data analysis.

**Results:**

Twenty patients were included in the study, and the majority of patients showed adherence (*n* = 14, 70%). The MC formulation used (*p* = .018), symptom relief (*p* = .001) and side effects experienced (*p* = .007) significantly influenced MC adherence. In addition to side effects experienced, findings for barriers to adherence were convergent or complementary for other medication-related factors, including the inconvenience of MC co-administration with food, cost and unpleasant taste.

**Conclusions:**

MC adherence is influenced by its effectiveness for symptom relief whereby appropriate MC formulation selection is crucial and should be determined by the indication (or symptom clusters). Social factors such as the views and experiences of close others had little bearing on MC adherence.

## Introduction

### Background/rationale

In Australia, there has been a rapid increase in prescribing medicinal cannabis (MC) since its legalisation in 2016 with pain, anxiety and sleep disorders being the leading indications [1, 2]. Although the evidence for its efficacy has been promising for other conditions, the variable quality of evidence remains a barrier to prescribing. The evidence has been considered to be of moderate quality for chronic pain and spasticity and low quality for chemotherapy-induced nausea and vomiting, weight gain in HIV, sleep disorders and Tourette syndrome [3, 4]. Nonetheless, MC is recommended for refractory symptom management, after standard supportive care therapies have been trialled and failed [5]. It is noteworthy that the use of cannabidiol (a single ingredient extracted from MC) for the treatment of refractory seizures of Dravet syndrome or Lennox-Gastaut syndrome is presently the only formulation and indications approved for Commonwealth government cost subsidy in Australia. Therefore, the full cost of MC is usually borne by the patient for other indications, which may pose a barrier to medication adherence and in turn potentially compromise patient outcomes. In addition to cost, concerns regarding addiction, psychoactive side effects and social factors such as support from friends and family or stigma have also been reported to impact patient preferences regarding MC use [6]. Consequently, this could lead to MC hesitancy and non-adherence and undermine therapeutic outcomes of treatments involving MC.

### Objectives

The objective is to investigate MC adherence rates and factors influencing real-world uptake of MC. Besides medication effectiveness, acceptability and affordability, social influences (such as fear of stigma) and perceived ability to control symptoms (or hopefulness) were hypothesised to influence MC use.

## Methods

### Study design

This study included a chart audit of demographic items and medication-related information (including MC formulation and indication) and a single, semi-structured interview with patients prescribed MC. The interview guide was developed based on the Theory of Planned Behaviour Framework (TPB) [7] which describes patient behaviour under the following TPB domains: *Attitudes*, *Subjective Norms*, *Behavioural Intention* and *Perceived Behavioural Control*. In general, *Attitudes* refers to the degree to which how favourably a person evaluates the behaviour in question (i.e. participant attitude towards MC usage as influenced by ‘medication-related factors’ or ‘personal factors’), *Subjective Norms* refers to the perceived social pressure to perform the behaviour (i.e. belief about whether most people approve or disapprove of their MC use; ‘social factors’), *Behavioural Intention* refers to the motivational factors that influences the behaviour (i.e. participant intention to use MC) and *Perceived Behavioural Control* refers to the sense of ease of performing the behaviour of interest (i.e. participant sense of ability to control symptoms experienced) and may be an indicator of ‘hopefulness’*.* Interviews were audio recorded and transcribed.

### Setting

The study was undertaken at a comprehensive cancer centre in metropolitan Sydney, New South Wales, Australia, where all eligible patients were invited to participate over a 6-month period (from 1/10/2022 to 31/3/23).

### Participants

Adult patients (aged 18 years or older) who were prescribed MC (oral oil) by a treating specialist physician of the centre were invited to participate in the study. The majority of the patients were instructed to adapt (self-titrate) their dose based on their symptoms and stage of their chemotherapy cycle and further advised that it could take between 20 min and 1.5 h to feel an effect. Patients were excluded if they were unable to consent for any reason, were highly dependent on medical care or experienced a cognitive impairment, intellectual disability or mental illness resulting in difficulties in understanding the information on the participant information statement.

### Variables

Participants who reported taking MC as recommended “All of the time” were classified as adherent, whilst those who used MC “Some of the time” or “None of the time” were considered non-adherent. Definitions were adapted from a similar study (involving medicines taken pro re nata) previously undertaken by Davies et al. (2013) [8]. Other information related to MC use was explored including indication, formulation, effectiveness, symptom control, side effects, cost, addiction, fear of stigma, preference for complementary, alternative and integrative medicine (CIM), any other concerns as well as their overall perception of their ability to control their symptoms/condition. Demographic data collected included age, gender, cultural background, spiritual/religious affiliation, socioeconomic status, highest education level attained, living circumstances, cancer type, presence of metastasis and performance status. Socioeconomic advantage and disadvantage was ascertained from the participant’s registered residential postcodes using the Australian Bureau of Statistics 2016 Index of Relative Socioeconomic Advantage and Disadvantage (IRSAD) [9] quintiles, whereby quintiles 1 and 5 correspond to the lowest and highest 20% local government areas in terms of socioeconomic advantage, respectively.

### Data sources/measurement

Demographic data and MC formulation used were extracted from the participant’s electronic medical record. Adherence to the prescriber’s recommendations, other information related to MC use (described above) and overall perception of symptom control were elucidated during the interview.

### Bias

Interviews were conducted by more than one investigator (UL, JG) to reduce interviewer bias.

### Study size

The study size was not pre-determined. Recruitment ended when empirical data reached thematic saturation, which was estimated to be between 9 and 17 interviews [10].

### Quantitative variables

Besides age which has been reported as continuous data, all other quantitative data was reported as categorical data.

### Statistical analysis

Descriptive statistical analysis was performed on all quantitative data. Tests of independence were performed on outcomes of interest as well as demographic characteristics to investigate any potential confounders. Mann–Whitney *U* and Fisher’s exact tests were utilised for continuous and categorical data, respectively, where a *p*-value < 0.05 was considered to be statistically significant.

### Qualitative data analysis

Responses which were related to factors that led to adherence were analysed inductively using NVivo^21^, which allowed themes and categories to emerge from the content. One author (UL) became familiar with the data by listening to the recording and reading the transcription repeatedly. Codes and themes were formed using a grounded theory approach as described by Glaser and Strauss [11] and reported under the most appropriate TPB domain. These were reviewed by a second author (JO).

### Triangulation of findings

Methodological triangulation of data obtained from medical records and patient interviews was employed to integrate findings. Findings from the two different methods were deemed “convergent” where findings from each method agreed, “complementary” where findings offered complementary information on the same issue or “dissonant” where findings appeared to contradict each other [12, 13].

### Reporting checklist

Reporting was undertaken in accordance with the STROBE Statement [14].

## Results

### Participants

Of the twenty-five patients approached, twenty-one patients met the eligibility criteria and were recruited to the study. One participant withdrew before the study commencement for personal reasons. The remaining 20 participants were included in the study and interviewed, guided by a schedule of questions developed using TPB domains as a framework (Table 1). The majority of participants (*n* = 14, 70%) were adherent and used their MC as recommended whenever required, whilst the remaining participants reported taking MC “Some of the time” when MC was needed for symptoms. None of the participants took their medications “None of the time”. The average interview duration was about half an hour.

**Table 1:** Study themes based on the TPB framework [7]

TPB domain	Interview themes and subthemes
Attitudes	Medication-related factors 1.1 MC effectiveness: symptom management 1.2 MC acceptability: side effects 1.3 MC viability: cost Personal factors 1.4 Preference for CIMs 1.5 Fears of dependence/addiction 1.6 Preference to drive
Subjective norms	Social factors 2.1 Others’ experience with MC use 2.2 Perceived approval of MC use from others
Behavioural intention	3.1 Observed adherence/non-adherence
Perceived behavioural control	Hopefulness 4.1 Overall perceived ability to control symptoms/condition

### Demographic characteristics

There were no statistically significant differences in demographic characteristics between the adherent and non-adherent groups (Table 2). Overall, the median age was 51 (IQR = 38–57) years and 59 (IQR = 45–59) years in the adherent and non-adherent groups, respectively. More than half of the participants in both adherent and non-adherent groups were females (*n* = 10, 71% and *n* = 4, 67%, resp.), had a primary cancer site involving the breast/gynaecological system (*n* = 7, 50% and *n* = 4, 67%, resp.) with metastasis (*n* = 9, 64% and *n* = 3, 50%, resp.) and were unable to maintain usual work/study (*n* = 9, 64% and *n* = 4, 66%, resp.). The majority of participants in both adherent and non-adherent groups lived with close others (*n* = 12, 86% and *n* = 4, 67%, resp.) in suburbs which were considered to be the “most advantaged areas” (i.e. IRSAD Quintile 5; *n* = 12, 86% and *n* = 5, 83%, resp.). Compared to non-adherent participants, more participants in the adherent group identified themselves as religious/spiritual (*n* = 8, 57% vs. *n* = 2, 33%) and completed education at a tertiary level or higher (*n* = 8, 57% vs. *n* = 2, 33%). However, neither characteristic was statistically significant. In both groups, few participants identified as CALD (*n* = 3, 27% vs. *n* = 2, 33%, resp.). Many patients were prescribed MC for more than one indication (*n* = 10, 71% vs. *n* = 4, 67%) with the most common in both the groups being insomnia (*n* = 9, 64% and *n* = 4, 67%, resp.) and pain (*n* = 8, 57% and *n* = 4, 67%, resp.) (Table 3).

**Table 2:** Demographic characteristics of adherent and non-adherent participants

Characteristic	Total (*N* = 20)	Adherent group (*n* = 14)	Non-adherent (*n* = 6)	*P*-value
Age, median (IQR) years	53 (40–59)	51 (38–57)	59 (45–59)	.312^a^
Gender, *n* (%)				
Female	14 (70)	10 (71)	4 (67)	
Male	6 (30)	4 (29)	2 (33)	1.000^b^
CALD, *n* (%)	5 (25)	3 (27)	2 (33)	.613^b^
Spiritual/religious, *n* (%)	10 (50)	8 (57)	2 (33)	.628^b^
Socioeconomic status: top 20% (IRSAD Quintile 5), n (%)	17 (85)	12 (86)	5 (83)	1.000^b^
Education level—tertiary or higher, *n* (%)	10 (50)	8 (57)	2 (33)	.628^b^
Living with close others, *n* (%)	16 (80)	12 (86)	4 (67)	.549^b^
Primary cancer site, *n* (%)				
1 Breast/gynaecological	11 (55)	7 (50)	4 (67)	
2 Colorectal	2 (10)	2 (14)	0	
3 CNS cancer	3 (15)	3 (21)	0	
4 Other cancers	4 (20)	2 (14)	2 (33)	.447^b^
Metastasis, *n* (%)	12 (60)	9 (64)	3 (50)	.547^b^
ECOG Performance Status, *n* (%)				
≥ 2/unable to maintain usual work/study	15 (75)	11 (79)	4 (66)	.613^b^

*CALD* culturally and linguistically diverse, *CNS* central nervous system, *ECOG* Eastern Cooperative Oncology Group [15], *IRSAD* Index of Relative Socioeconomic Advantage and Disadvantage [9]

^a^Mann-Whitney *U* test

^b^Fisher’s exact test

**Table 3:** Reported indications for MC

Indications	Total	Adherent	Non-adherent	*P-*value^a^
Anxiety	8 (40)	6 (43)	2 (33)	1.000
Appetite	3 (15)	3 (21)	0	.521
Depression	1 (5)	1 (7)	0	1.000
Fatigue	2 (20)	2 (14)	0	1.000
Insomnia	13 (65)	9 (64)	4 (67)	1.000
Pain	12 (60)	8 (57)	4 (67)	1.000
Nausea	10 (50)	9 (64)	1 (17)	.141
Relaxation	2 (10)	1 (7)	1 (17)	.521

^a^Fisher’s exact test

### Medication-related factors impacting adherence

Adherent participants were more likely to receive a balanced MC formulation (i.e. equals parts CBD (cannabidiol) and THC (tetrahydrocannabinol)) compared to non-adherent participants who were more likely to receive a CBD-only/dominant formulation (*n* = 11, 79% vs. *n* = 1, 17%, *p* = 0.018; Table 4). There was also a significant difference in experience with MC use particularly in terms of effectiveness and adverse effects, experienced between the study groups. Compared to non-adherent participants, more adherent participants experienced symptom relief “all of the time” (*n* = 13, 93% vs. *n* = 1, 17%, *p* = 0.001) and less adverse effects (*n* = 2, 14% vs. *n* = 5, 83%, *p* = 0.007) when MC was used. Few participants reported concerns about MC costs in both groups (*n* = 4, 29% and *n* = 0, 0%).

**Table 4:** Experience with MC

MC-related experience, *n* (%)	All (*N* = 20)	Adherent (*n* = 14)	Non-adherent (*n* = 6)	*P*-value^a^
Formulation				
CBD only/dominant	8 (40)	3 (21)	5 (83)	
Balanced	12 (60)	11 (79)	1 (17)	.018*
Over the past week, MC was used at least once	15 (75)	10 (71)	5 (83)	1.000
Symptom relieved “all of the time”	14 (70)	13 (93)	1 (17)	.001*
Any side effects	12 (60)	2 (14)	5 (83)	.007*
Driving concerns				
Driving frequency, ≥ 3 days/week	15 (75)	10 (71)	5 (83)	1.000
Driving importance	11 (55)	8 (57)	3 (50)	1.000
Driving impairment	7 (35)	5 (36)	2 (33)	1.000
Knew someone close who:				
Used MC	10 (50)	8 (57)	2 (33)	.628
Had a positive experience	9 (45)	7 (50)	2 (33)	.642
Influenced patient choice	6 (30)	4 (29)	2 (33)	1.000
Cost concerns	4 (20)	4 (29)	0	.267
Fear of addiction	2 (10)	2 (14)	0	1.000
Preference for MC/CIM	7 (35)	4 (29)	3 (50)	.613

Statistically significant differences (*p*-value < .05) highlighted with an asterisk (*)

^a^Fisher’s exact test

### Patient-related factors impacting adherence

The minority of participants in both adherent and non-adherent groups had a preference for CIM (*n* = 7, 35% and *n* = 4, 29%, resp.) or feared addiction to MC (*n* = 2, 10% and *n* = 2, 14%, resp.). Driving-related concerns were also similar between both groups. At least half of the participants in both groups considered maintaining the ability to drive as “important” (*n* = 8, 57% and *n* = 3, 50%, resp.), with a majority driving at least 3 days per week (*n* = 10, 71% and *n* = 5, 83%, resp.) and not experiencing driving impairment.

### Social factors

More participants in the adherent groups knew someone close who used MC compared to the non-adherent group (*n* = 5, 58% vs. *n* = 2, 33%, *p* = 0.628) and had a positive experience (*n* = 7, 50% vs. *n* = 2, 33%, *p* = 0.642), but neither were found to be statistically significant. Furthermore, few patients’ own MC use was influenced by close others in either group (*n* = 4, 29% and *n* = 2, 33%).

### Symptom control and hopefulness

Compared to the non-adherent group, more participants in the adherent group considered their symptoms to be well-managed overall (*n* = 9, 33% vs. *n* = 2, 33%, *p* = 0.603) and were hopeful that their symptoms were controllable in general (*n* = 9, 64% vs. *n* = 1, 17%, *p* = 0.642), but this was not found to be statistically significant.

### Interview results

The main reasons for potential or actual non-adherence reported were medication and/or patient-related barriers. Medication-related factors included lack of belief in the effectiveness or experiencing a negative response to MC, inconvenience of needing to take MC with food, MC cost and unpleasant taste of the oil formulation. Patient-related factors included driving impairment or fear of legal repercussions of concomitant use whilst driving, forgetfulness and feeling too unwell to use MC. Triangulation of findings from medical records and interviews is summarised in Table 5.

**Table 5:** Triangulation of qualitative and quantitative results by subthemes

TPB domain	Themes	Subthemes	Quotes	Mixed-methods triangulation
Attitudes	Medication-related barriers	1.1 MC effectiveness: symptom management	Facilitators *“… with the anxiety, the depression, the nausea, the sleep… it was helping my appetite… It just helped with absolutely everything…”* [P7]	Convergent
		1.2 MC acceptability: side effects	Facilitators *“You get a really good night sleep… but without it being that grogginess the next day”* [P4] Barriers *“I don’t like to feel sleepy during the day.”* [P9] *“I don’t actually welcome the high, particularly I just want symptom control.”* [P15]	Convergent
		1.3 MC viability: cost	Barrier *“It's quite an expensive product; that will be the only barrier…”* [P3] *“I probably would… put it off a lot longer too…if I was to buy it again at the original price… I'd wait a long time before I get another bottle.”* [P12]	Dissonant
		Other	Troublesome dosing requirements *“Sometimes I really didn’t feel like anything to eat, but then I’d have to eat a piece of cheese to have my cannabis oil… that just was a bit of an annoyance.”* [P11] Unpleasant taste *“I do try that but sometimes… it actually tastes quite disgusting.”* [P9]	Complementary
	Patient-related barriers	1.6 Preference to drive	Barriers *“The only thing that I was just concerned about, like starting up driving. That's all… I'm hoping that it [MC] will be out of the system when I drive.”* [P7] *“… at the moment, it's effective and I only stopped because I have to drive; that's the only reason.”* [P12]	Dissonant
		Other	Forgetfulness and deteriorating health *“I’d actually forgotten to take it.”* [P16] *“I’m just really tired… So I might just [take MC] on some random nights, through either not forgetting or just thinking it’s too late.”* [P10] *“I think that would be the only time I wouldn’t take it; when I’m just too sick and out of it.”* [P5]	Complementary

## Discussion

### Key results

In this study, advanced cancer diagnoses were observed in both groups, whereby the majority had metastatic disease and were unable to maintain usual activities such as work or study. The most common indication for which MC was prescribed in this study was insomnia (or sleep disturbance) and pain, with almost half of patients in both groups experiencing both as a symptom cluster, defined as two or more symptoms that are related to each other and that occur together [16]. Significantly more patients in the adherent group were prescribed a balanced MC formulation (rather than CBD-dominant MC formulations) and found MC to be effective for symptom relief. These findings are largely attributed to the fact that a balanced MC formulation provides better pain control among cancer patients [17], and given the bi-directional effect between pain and sleep [18, 19], it is anticipated that better pain control will correspond to better sleep (and vice versa). This emphasises the advantage of using MC for the treatment of symptom clusters and the importance of appropriate MC formulation selection with respect to indication. Furthermore, the favourable pharmacotherapeutic profile of MC, namely, its more tolerable side effects (e.g. decreased “hangover” or next-day residual effect in contrast to other medications such as benzodiazepines) was also highlighted as favourable by patients. Not surprisingly, a greater proportion of the adherent group (about double) also considered their symptoms to be “well-managed overall” and were more hopeful, believing their symptoms could be controlled. This however was not statistically significant and likely a result of insufficient statistical power (< 20%, ⍺ = 0.05) to detect significance in hopelessness with regards to pain experienced as described in published data [20].

Non-adherent patients reported inconvenience of MC co-administration with food which they believed to be a dosing requirement. Administration with fatty food increases the bioavailability of MC (and impacts its therapeutic effect) [21, 22], and hence, consistency of MC administration with respect to food is advised. This means that co-administration without food may also be considered (if it is consistent) for patients who experience nausea/vomiting or other issues affecting their appetite. The other medication-related barrier mentioned was the unpleasant taste of the MC oil which was administered sublingually in this study. This issue may be alleviated through consideration of alternative delivery methods that do not involve oral oil ingestion (or oro-mucosal sprays) such as oral capsules, pulmonary (vapourised or inhaled) or topical routes [23].

The impacts of MC on one’s ability to drive, namely, physical impairment and legal prohibitions, were also reported as reasons for non-adherence. Here, the prescriber could recommend not taking MC if driving, and as a result, some patients would take “breaks” or “holidays’ from MC which increased the risk of negatively impacting symptom management but enabled legal driving. As with other prescription medications which may impair alertness and cognition, it has been suggested that the same precautions should be taken by patients affected by MC [24]. A review of legal frameworks for driving under the influence of drugs, which currently criminalises the presence of THC in a driver’s bodily fluids with no regard for the level of impairment, may consider ‘medical exemptions’ for MC as an alternative approach [25]. General unwellness and cognitive impairment leading to forgetfulness were previously overlooked barriers resulting in non-adherence. Although MC use may be a contributing factor, these symptoms are not unique to MC use or our study setting. Forgetfulness resulting in unintentional non-adherence has also been observed in populations with other serious conditions such as breast cancer, multiple sclerosis and human immunodeficiency virus [26–28]. Assessing and addressing any cognitive (including memory) issues at treatment commencement, as well as utilising reminders and avoidance of contributing substances where possible, has been advised here.

Patients were not deterred by the potential for addiction to MC, although some mentioned disliking the psychoactive side effects or ‘high’ feelings associated with its use. Overall, adherence was not influenced by the experiences or opinions of close others who also had experience with MC use. On the other hand, patients were often more inclined to trial MC as a potential therapeutic product if encouraged by their physician. Support by physicians for trialling all therapeutic options available is well known in the supportive and palliative care setting, particularly in the face of suffering and limited options for refractory symptom management. This is commonly seen in settings involving refractory cancer pain, advanced life-limiting or complex symptom management in palliative care settings and refractory childhood epilepsy [29–31].

### Limitations

Patients were predominantly from areas of high advantage (i.e. from the top IRSAD Quintile); hence, the affordability and therefore viability of MC therapy may not be generalizable to people from less advantaged communities. In fact, uncertainty regarding the impact of the cost was highlighted by a ‘dissonant’ outcome resulting from the triangulation of findings with respect to the impact of MC cost. Most patients in this study also identified as Caucasian and therefore may not be reflective of the sentiments of people in the wider community, particularly those that identify with a CALD background whereby fear of stigma and addiction, which was not apparent in this study, may be a significant barrier.

### Interpretation

Overall, adherence and optimization of therapy with MC were primarily driven by factors related to the medication and patient. Medication-related factors can be addressed in a relatively straightforward manner, namely, through a rational selection of appropriate MC formulations (namely, cannabinoid and terpine profile, CBD:THC ratio and mode of drug delivery) and appropriate patient counselling. State legislation which currently prohibits driving with THC use lags behind clinical practice and undermines patient therapy and/or quality of life. Hence, policymakers are urged to carefully review this legislation and implement considered amendments. Non-adherence due to fear of addiction or stigma with MC use was unfounded in this study population.

### Generalisability

In the management of common symptoms in the advanced cancer setting including pain and insomnia, MC shows promise as an effective, acceptable and potentially affordable therapy. In comparison to other medications such as benzodiazepines, it has a favourable side effect profile and may be particularly useful for the treatment of symptom clusters, alleviating multiple symptoms, which may reduce the likelihood of polypharmacy and its associated risks.

### Other information

No funding was received by any of the authors for the undertaking of this work.

## Data Availability

No datasets were generated or analysed during the current study.
